# The learning environment on a student ward: an observational study

**DOI:** 10.1007/s40037-019-00538-3

**Published:** 2019-10-08

**Authors:** Anna Dyar, Hanna Lachmann, Terese Stenfors, Anna Kiessling

**Affiliations:** 1 Department of Clinical Sciences, Danderyd Hospital, Karolinska Institutet, Stockholm, Sweden; 2 Department of Learning, Informatics and Ethics, Karolinska Institutet, Stockholm, Sweden; 3 The Swedish Red Cross University College, Stockholm, Sweden

**Keywords:** Observation, Professional education, Peer learning, Student ward

## Abstract

**Introduction:**

Worldwide, a growing number of healthcare students require clinical environments for learning. Some wards have become adapted ‘student wards’ to meet this demand. Benefits have been reported from the students’, supervisors’ and patients’ perspectives. There is no definition of a student ward, and little research on what the term means. A deeper understanding of the characteristics of student wards is needed to support their use. The aim of this study is to describe what characterises the learning environment on one student ward.

**Methods:**

An ethnographic approach was used for an observational study on a student ward in a hospital in Sweden. Student nurses, supervisors and others on the ward were observed. Field notes were thematically analysed.

**Results:**

Four themes were identified: ‘Student-led learning’ described students learning by actively performing clinical tasks and taking responsibility for patients and for their own learning. ‘Learning together’ described peer learning and supervision. ‘Staff’s approach to learning’ described personalised relationships between the students and staff and the build-up of trust, the unified inter-professional approach to teaching, and the supervisors’ motivation for teaching and for their own learning. ‘Student-dedicated space’ described the effect of the student room on the learning environment.

**Discussion and conclusions:**

This study describes the characteristics of a student ward that centred around a community of practice that shared a view of learning as a priority, allowing staff to provide clinical care without compromising students’ learning. This qualitative study at a single centre lays the groundwork for future research into other student wards.

**Electronic supplementary material:**

The online version of this article (10.1007/s40037-019-00538-3) contains supplementary material, which is available to authorized users.

## What this paper adds

Student wards are becoming increasingly important clinical settings for student learning. Although many studies evaluate their outcomes, there is little knowledge of what a student ward actually is. This study used an ethnographic approach to holistically study a student ward and to provide a rich description of the learning environment. This is the first step towards exploring the characteristics and inter-relatedness of other student wards, to support their development and use in future medical education.

## Introduction

As pressure on service provision in healthcare rises, the numbers of students are increasing to meet the demand for qualified healthcare professionals, making education in the clinical environment a growing challenge. It is important for the future of healthcare to ensure sustainable settings for learning in the clinical environment without compromising the quality of patient care. Development of professional communication and collaboration competences is crucial for patient safety [1].

Whilst many students continue to learn in regular clinical settings, some settings have been specially adapted to student learning [2–6]. These have been referred to in the literature as: clinical education wards; interprofessional training wards [7–10]; dedicated education units [6, 11–15]; student training wards [5]; and student-run clinics in primary care [16]. A unified term is lacking, so we use the descriptive term ‘student ward’ to encompass all clinical settings adapted specifically to students. Student wards have various purposes, such as to promote interprofessional learning [5], problem-based learning [17], or to allow increased numbers of students [13]. Student wards have been adapted to students of nursing, medicine, occupational therapy, physiotherapy, social work etc. [5, 13, 18–20]. In Sweden, student wards are being set up to create high-quality learning environments for an increased number of students and to create a good working environment for their supervisors. Whilst student wards may vary in their purpose and set-up, they share the common feature of permanent adaptations to accommodate students.

Previous studies have indicated benefits of student wards, from the perspective of students [6, 16, 21], supervisors [9, 16, 17] and patients [16, 22]. Some focussed on evaluating outcomes such as interprofessional practice [5, 19, 21] or clinical learning [8, 23]. However, little research exists on what the clinical learning environment is like. The clinical learning environment has attributes including the physical space, psychosocial and interaction factors, the organisational culture, and teaching and learning components [24, 25], and has important effects on achievement of learning outcomes [24, 26]. Although differences in the learning environment are bound to exist between many diverse student wards, there could be characteristics that they have in common that distinguish them from traditional wards. The heterogeneity of student wards and the generalisability of findings from one ward to another have not been addressed in the literature.

Following the reported benefits of student wards, new student wards have increasingly been set up in Sweden. However, without the knowledge of preceding student wards and their clinical learning environment, new wards rely on informal contact with staff from existing wards to inform them how their new ward can be set up, as published literature on student wards is so far limited to evaluations of their success. Moreover, those without access to informal advice from colleagues have no guidance. As evidence of the success of student wards is increasing, knowledge of what it means to be a student ward should increase in order to best inform and guide future wards so that they can share experience from their counterparts without wasting time and resources.

The identified previous observational studies of student wards have been limited to the patients’ perspective [10], the supervisors’ perspective [9] and a case study on interprofessional learning [19]. To our knowledge our study is the first to investigate the clinical learning environment of a student ward holistically, using an ethnographic approach. The aim of this study is to describe what characterises the learning environment on one student ward.

## Methods

### Study design

This study used an ethnographic approach to explore and understand social settings and processes, a methodology which is increasingly being applied in the field of medical education [27, 28]. Learning was viewed according to Wenger’s social theory of learning, where learning is social and comes largely from of our experience of participating in daily life [29] rather than an individual and discrete activity.

### Study setting

The student ward was on an acute medical ward in a teaching hospital in Stockholm. It was set up by the nurses in charge of the ward together with the school of nursing in February 2015, with adaptations for student nurses, although medical students were also present. Nurse supervisors with an interest in teaching were recruited to the ward. The student ward had six patient beds, and another six-bed regular ward adjoined the student ward. A pair of student nurses and one supervisor cared for the patients on the student ward during weekday day and evening shifts.

### Participants

The student nurses in term three (T3) and term six (T6) were present for 5 and 6 weeks respectively. Nurse supervisors, doctors, healthcare assistants, other healthcare professionals, patients, relatives and visitors were also observed. All participants were informed about the study and gave oral consent.

### Data collection

Daily life on the ward was observed over a 6-month period (April to September 2017). An observation guide (Electronic Supplementary Material, Table S1) was developed based on the research question, one of the author’s ethnographic observations of clinical settings from previous experience [30, 31], and refined after a pilot observation. Field notes were taken contemporaneously and transcribed immediately after the shift. Participant data were anonymised. The observer (first author), a doctor by profession, was introduced to the participants in the role of a researcher. The observer wore hospital clothes and a name tag. The observer did not work on the ward or at the hospital, had no connection with the participants and was non-participatory.

The intended observation period was 100 h, according to previous similar observation studies [9, 31]. The factors taken into account for the length of the observation were: the broad nature of the aim; the specificity of participants’ behaviour with regard to the student ward; and the small number of previous studies which can be used as a baseline establishedtheory [32].

Informal and spontaneous short informal questioning of participants by the observer took place in quiet locations when there was no other activity taking place and with only the participant(s) and observer present, aiming to gain a deeper understanding of the observed actions and participants’ thoughts and reasoning. The answers were recorded as field notes with verbatim quotations.

All student nurses on the student ward were approached by the researcher and given the opportunity to submit an audio diary at the end of a shift, for as many shifts as they chose. Their guidance was to reflect freely (as they would in a diary) on anything to do with their learning on the student ward that day. Audio recordings were made by students themselves and then submitted securely to the researcher. Audio recordings were transcribed and included in the analysis.

### Ethics

Ethical approval was received from the regional ethical committee in Stockholm (Dnr 2016/2524-31/2).

### Data analysis

A preliminary analysis was performed simultaneously with the data collection to refine the observation guide, detect areas needing further investigation and to have a continuous overview. The observations were concluded when the research team felt that the data collected could answer the research question, guided by the richness of the data as determined by the large amount of meaningful, relevant and illuminating data collected during this time and the repetitiveness of the observed phenomena [32]. After all data had been gathered, a thematic analysis of the data was performed according to the description by Braun and Clarke [33], with an inductive approach. Thematic analysis is a qualitative analysis method for identifying, analysing and reporting patterns in the data, as well as interpreting various aspects. It was chosen because of its flexibility in combining diverse forms of data. After familiarisation with the data by reading the field note transcripts, initial codes were generated by the first author to identify interesting features of the data systematically. The codes were then collated into subthemes and themes. These themes were discussed among all authors until a consensus was reached and were refined against the initial codes, then defined and named to give a description of the meaning behind them.

The findings of the study were presented to and discussed with the staff on the student ward. The findings were confirmed by their unanimous agreement with the author’s interpretation of the observations.

## Results

The observations covered 17 different shifts, each lasting about 5 h, approximately 85 h in total. About 310 events were observed, including ward rounds, handover meetings, board rounds, clinical tasks, procedures, phone calls, case discussions and informal conversations. There were 31 instances of short informal questioning and three audio reflections. The participants are described in Table 1.

**Table 1:** Participants

Participants	Total
Nurse supervisors	7
Student nurses: total	12
– Term 3	5
– Term 6	7
Doctors	6
Medical students	3
Healthcare assistants	3
Healthcare assistant students	4
Other staff: – Biomedical scientists – Bed coordinator (registered nurse) – Psychologist – Social worker	5

Four themes were identified that characterise the learning environment on the student ward: student-led learning, learning together, staff’s approach to learning, and student-dedicated space. Examples of field notes under subthemes can be found in the Electronic Supplementary Material (Table S2).

### Student-led learning

Student nurses actively performed nursing tasks rather than passively watching their supervisor. Students were by default expected to be the patients’ primary caregivers on the ward and expressed feelings of ownership of patients’ care. The learning activities were based on clinical events, never preselected. Students were responsible for task allocation and sought ways of meeting their learning needs by asking many more questions than they were asked by the staff. The supervisors acted more like guides, role models or helpers than like teachers.

### Learning together

A pair of students shared one supervisor and often shared responsibility for the same patients. Students were often physically located in the same room (patient room, medicines room or student room). Frequent interactions between the pair occurred in the form of questions, performing a task together, and solving clinical or practical problems, and students were encouraged to seek help from the supervisor after they had first tried to resolve the problem together. Near-peer shifts were scheduled in which a T6 acted as the T3’s supervisor, and were perceived by both students as providing unique perspectives and opportunities. During these shifts, the T6s posed many questions to T3s, gave feedback and created opportunities for them to practise practical skills. The nurse supervisors viewed the supervision of multiple students as challenging at the start due to the dynamics of their personalities and interactions as well as to their different strengths and weaknesses. However, the supervisors felt it was beneficial in the long run for their learning from one another, their eventual self-sufficiency and their development of teamwork. All supervisors attended general supervisor courses and weekly supervisor meetings to discuss students as well as to reflect on supervisor issues and peer support for supervising peer learning.

### Staff’s approach to learning

The supervisors and staff became acquainted with the students, adapting their approach according to their individual learning goals, previous experience, strengths and weaknesses, and learning styles. Students were addressed by their first name by both their supervisors and other staff. Students questioned the staff, showing no barriers of hierarchy, even expressing dissatisfaction with the answers (see Table S2, Electronic Supplementary Material). The personalised approach enabled the development of trust over time between the student and the supervisor. This was demonstrated by students being given increasing independence and increasingly frank feedback from supervisors, and even conflict situations (see Table S2, Electronic Supplementary Material) were perceived as constructive by the student and supervisor.

Learning was a central part of daily life across all professions. Interruptions to the normal flow of activities for educational reasons during multi-professional meetings occurred regularly. Pauses for teaching were actively created as part of the normal ward activities, both by nurse supervisors and staff without supervisor roles. The observer reflected that this was a well-functioning ward which the staff were proud of, and that the students felt lucky to have a clinical placement on the student ward.

Student nurses interacted with doctors, healthcare assistants and biomedical scientists, as well as with students of other professions (medical students and healthcare assistant students). However, unlike the ‘learning by doing’ approach with nurse supervisors, students’ (both nurse and medical students) interaction with other staff involved answering questions, observing practice and theoretical discussions. There were no formal adaptations of the student ward for medical and healthcare assistant students, and although they were unaware that the ward was adapted to students, they described the ward as having an especially ‘pedagogical atmosphere’.

Supervisors described their supervision role as an important and fun part of their job from which they derived satisfaction, and they stated that teaching was the motivating factor for them working on the student ward. Learning was not limited to students, and staff regularly paused to explain an aspect of their clinical work to their colleagues beyond what was necessary for their communal care of the patient. Staff attended educational courses, and the observer reflected that learning was seen as a continuous process for all professions at all stages.

### Student-dedicated space

The student room was regarded as a central meeting point where students and supervisors could easily find one another on the busy ward. Even though a nurse office was located nearby, staff would automatically go to the student room for handover meetings when the students were present. Students commanded ownership of the room: medical equipment (blood pressure cuffs, stethoscopes, pulse oximeters, thermometers) and the medicines trolley were in the students’ territory and by extension it was their responsibility to perform the task or delegate it. Computers in the student room were exclusively for student use, so students performed all note-taking and looking-up during meetings. When questioned about their experience on the student ward compared to previous ward placements, the T6s responded that a key feature was having their own private space, where they could take their time to complete tasks without feeling rushed or feeling that they were taking up a computer and preventing someone from doing their job. The students took on more leadership roles, made more decisions and asked more nuanced questions (see Table S2, Electronic Supplementary Material) in the student room compared to similar meetings in the nurses’ office.

## Discussion

A student ward was observed, and the findings described themes that characterise the clinical learning environment. The theme ‘student-dedicated space’ alludes to features of the physical space. ‘Learning together’ and ‘staff’s approach to learning’ allude to many psychosocial and interaction factors; ‘student-led learning’ and ‘staff’s approach to learning’ have features relevant to the organisational culture, and all four themes relate to the teaching and learning components. These themes provide a description based on what actually was observed happening on the ward, in addition to participants’ perceptions [28, 34, 35]. The study encompasses many different perspectives, uniting aspects previously observed in isolation.

The observation period covered a variety of patients, workloads, supervisors, staff and students. However, the characteristics identified in the four themes remained consistent. Using Wenger’s social theory of learning [29], these themes can be seen as characteristics of the community of practice. This community of practice characterised the student ward: the staff and students had a shared view of learning as a priority, and staff had a united passion for supervision and somewhat automatically centred their practice around the students.

‘Student-led learning’ referred to students’ active learning by participating in clinical practice rather than passively following or observing their supervisors. This phenomenon has been described in the literature: ‘active engagement’ on a student ward was described in a previous study as giving an experience of authenticity that forms the core of student learning [36]. A hands-off supervisor’s role has been described as giving students a supported but participatory role in practice, which is a core condition of workplace learning [37]. The students in this study were active not only in patient care but were responsible for their own learning. This is also in line with a previous observational study on a student ward where supervisors viewed their role as facilitating and allowing students independence [9].

Clinical events guided students’ activities, rather than supervisors’ directions, so that students’ activities closely mimicked the realities of working as a nurse. This contrasts with a previous case study of an interprofessional training ward in a nursing home, where students were excluded from some activities and had arranged learning situations instead, which they perceived as make-believe and unrealistic [19]. The difference in the ‘student-led’ approach between the student wards could be explained by the nursing home setting, or its dual focus on interprofessional practice. However, differences are not surprising given there is no uniformity among student wards. A community of practice evolves through changing relationships and participation and engagement with one another and each other’s work, and therefore could turn out very differently in different student wards despite common aims.

The practice of peer learning was a key adaptation of this student ward. Peer learning can be defined as ‘people of similar social groupings who are not professional teachers helping each other to learn and learning themselves by teaching’ [38], and is increasingly implemented in medical education [39]. Learning together was previously observed even on a ward with no explicit framework of peer learning [9]. Students described the similar advantages of peer learning as those previously reported in the literature: learning through teaching peers increasing and validating their knowledge [40], building confidence [41]; preparing students for their future teaching role [42–44]; increased learning through social and cognitive congruence [40, 45], where the teacher and students share a similar knowledge base, allowing the teacher to explain concepts at an appropriate level [46]. On this student ward, peer learning was enabled by two students timetabled simultaneously on the ward, sharing a supervisor, and co-scheduling of students from different terms and allocating them explicit supervisory roles. The community of practice supported the supervision of peer learning by dedicating a weekly meeting to support the supervisors in developing their supervision skills. The ability to teach is a requirement for registered nurses [47], yet student nurses in their final term report that, during their whole nursing education, it was only on the student ward that student nurses had the opportunity to practise teaching. Student wards therefore have the potential to fill this important gap in nurse education.

In contrast to many student wards adapted to interprofessional learning [5, 9, 21, 48], this student ward did not include medical and other students in its set-up, although they were present on the ward. It is notable that although medical students were observed participating in learning activities with student nurses, they were unaware that they were on a student ward. A previous study emphasises that the learning environment for students of different professions is different even if they are present simultaneously on the same ward [30].

The ‘staff’s approach to learning’ described how staff integrate the students into the community of practice. A student’s role has been described as starting as a newcomer with peripheral participation, then progressing to fuller participation through having legitimate access [49]. ‘Access’ to active involvement in a student ward is explicitly accepted; however, previous studies have shown that this alone is insufficient for full participation [19]. In this study, the personalised approach between the staff and students aided in the transition to being an active participant in the community of practice. Each student was, at their own pace and according to their individual needs, interests and personalities, transitioned into being the primary caregiver, the ‘real’ nurse on the ward. Following the build-up of mutual trust, students became legitimate participants, increased their questioning and initiative, and dared to express conflicting opinions.

Due to the busy nature of a clinical learning environment, clinical care is often prioritised over student learning in regular wards [50], whereas the community on this student ward viewed learning as being integrated into their clinical work. This is in line with a previous observational study which found that supervisors on a student ward viewed supervision and patient care as equally important and interrelated rather than separate tasks [9]. In this study, while balancing teaching and patient care was a challenge, the community of practice supported this dual role. Staff also recognised and prioritised their own continued learning through their clinical work and did not see learning as being confined to the students, making supervision a natural extension of their own way of practice. It is important to note the contribution of organisational factors to enabling such a community; the student ward was set up with the head nurses due to their desire to support student education, and the recruitment of nurse supervisors was performed targeted to this specific role. This could be an important precondition for a student ward to maintain a community of practice that supports student learning.

The student-dedicated room was an important component of the clinical learning environment, both the physical space, the equipment located in it, as well as the atmosphere in the room. There are no descriptions of the physical space of student wards in the literature, other than specification of the number of beds present. The permanency of the student room, and its explicit purpose, served as a constant reminder of the purpose of the student ward and could be seen as a statement of its commitment to students. Aside from the reported practical facilitations of the room, the atmosphere created there had an effect on the behaviours of the students and the staff. It was interpreted that their type of questions (Table S2, Electronic Supplementary Material), and thus their underlying expectations and attitudes were more in line with deep learning compared to any other locations on the ward. Deep learning ‘is characterised by examining new facts and ideas critically, tying them into existing cognitive structures and making numerous links between ideas’ [51]. Although the presence of the room alone is insufficient to create a facilitating atmosphere for learning, it was a necessary component for deep learning that could be transferrable to other student wards.

This study characterised the clinical learning environment on one student ward. These characteristics could be a consequence of its adaptations to being a student ward, although there may be features in common with traditional settings. Whether the findings are transferrable to other student wards is uncertain; indeed, the level of heterogeneity in the different student wards and the lack of any established term or definition is one of the problems this study sets out to address. Future research is needed to establish whether the variability between different student wards allows any unified description of student wards’ characteristics to exist. There is great heterogeneity also of traditional learning environments, making the relationship between student wards and regular wards difficult to determine. Further studies to establish whether the characteristics of a student ward that are linked with positive learning outcomes can be employed on a regular ward are important not only for setting up new student wards but for creating positive clinical learning environments even in regular clinical settings.

Whilst various outcomes of student wards have been evaluated previously in the literature, the relationship between observed characteristics of this student ward and the previously reported outcomes from the student, supervisor, patient and organisation’s perspective has not been established. This study provided information on the characteristics of a student ward as an increasing number of new student wards are set up. However, how these characteristics are achieved and the preconditions for their success are important questions to be researched before there can be any practical application of the results of this study in clinical practice.

### Limitations

There is an inevitable effect of the observer’s presence on the participants’ behaviour, and an extended observation period aimed to minimise this. The researcher’s prior knowledge and pre-understanding of clinical wards inherently introduced subjectivity into the interpretative analysis of the observational material, and the observer was not naïve as in traditional ethnographic research [52]. These biases were tempered by the observer having no previous connection to the participants or the student ward. The observer was a doctor by profession, giving an understanding of the medical context, but without the specific nursing background of most of the participants. This had the effect of making the participants feel less like they were being evaluated, and minimising the bias of personal experience of the observer, but limiting profession-specific nuances, which the co-author, who is a nurse, strove to compensate for. The observer not being a nurse also minimised both overvaluations of observed features that were different to expectations and disregarding of factors that are taken for granted. Although participants commented that they soon forgot that the observer was there, potential effects on the participants’ behaviour are likely to have affected the observations to some degree.

The informal questioning and audio diaries gave a degree of methodological triangulation. The audio diaries allowed students to express themselves unimpeded by aversions to speaking face-to-face with an interviewer. However, the small number of recordings limited their use.

This was a single-centre study, and the number of student wards with the same set-up regarding profession of students, type of clinical setting, and other organisational features is limited. There may be cultural or organisational features that limit transferability to student wards in other countries or organisational/political circumstances. This student ward was set up for student nurses, and it is questionable whether the findings involving student nurses can be applied to medical students and students of other professions, due to the differences in the nature of the content of the learning, aims, and the organisation of the curriculum and clinical placements. A comparison between characteristics of a student ward and a traditional ward was outside of the scope of this study, and a further study is planned involving multiple student wards and traditional ward counterparts.

## Conclusion

This study describes the characteristics observed on a student ward: student-led learning with students learning together, a personalised and motivated approach by the staff and supervisors, and facilitation of the student-dedicated space. This qualitative study at a single centre lays the groundwork for future research to investigate other student wards and how these characteristics affect student learning. These findings could aid in the future use of student wards for medical education.

## Caption Electronic Supplementary Material

*Table S1* Observation guide

*Table S2* Results: themes, subthemes and example of field notes
