# Supplementary material for: The learning environment on a student ward: an observational study
Source: Perspect Med Educ. 2019 Oct 8;8(5):276–83. doi: 10.1007/s40037-019-00538-3 (PMC6820594; doi:10.1007/s40037-019-00538-3)
Supplement: Supplementary file 1 — Table S1 Observation guide [file 40037_2019_538_MOESM1_ESM.docx]

**Table S1** Observation guide

| Event description |
| --- |
| Time, date, duration |
| Atmosphere, staffing, conditions |
| Location of event |
| Participants present |
| Diagrams of participant placement, room plans. |
| What happened? |
| What was said? |
| What was done? |
| Notable things that did not happen |
| Key direct quotations |
| Researcher’s reflections |
