# Supplementary material for: The learning environment on a student ward: an observational study
Source: Perspect Med Educ. 2019 Oct 8;8(5):276–83. doi: 10.1007/s40037-019-00538-3 (PMC6820594; doi:10.1007/s40037-019-00538-3)
Supplement: Supplementary file 2 — Table S2 Results: themes, subthemes and example of field notes [file 40037_2019_538_MOESM2_ESM.docx]

**Table S2** Results: themes, subthemes and example of field notes

| **Theme** | **Sub-theme** | **Example from observation field notes** |
| --- | --- | --- |
| Student-led learning | Students learning by doing | *A healthcare assistant asked a T6 directly about a patient’s progress directly even when the supervisor was present. Student: ‘He is my patient’.* |
|  | Supervisors’ standing-back role | *A student connected a cardiac monitor for the first time. The supervisor described where to put the leads but did not touch the equipment.* |
|  | Students’ responsibility for their own learning | *The pair of students divided the patients between themselves according to their learning needs. They reported back to the supervisors, who asked for clarifications but were never observed leading such encounters.*  *During a set of students’ placements on the ward, a total of 49 clinical knowledge-based questions were observed between students and staff. Of these, 42 were asked by the student to staff, 7 by the doctor to the students, and none by the nurse supervisor.* |
| Learning together | Peer interactions for learning | *Students primarily discussed their questions between themselves before asking their supervisor.*  *Questioning T3 students: They described their supervision by T6s as inspirational, enabling them to see how far T6 students had progressed and get an idea of the level they were expected to reach in three terms’ time.* |
|  | Scheduling for near-peer learning | *There were shifts where T3 and T6 students were scheduled simultaneously with the specific intention of T6 practising near-peer supervision. T6 students stated that the student ward was the only clinical setting where they had ever had teaching practice.* |
|  | Supervising peer interactions | *The supervisor was present but busied herself with another task while observing the T6 supervising the T3 preparing an intravenous infusion.*  *Supervisor questioning: ‘Supervising multiple students is challenging when they have different needs or doing different things, but beneficial when students can support and learn from one another’.* |
| Staff’s approach to learning | Personalised relationships and the build-up of trust between the students and staff | *The students were addressed by their first names by all members of staff, and their names were written above patient beds as key caregivers.*  *A student repeatedly questioned the doctor about the reason a patient had been admitted and was only satisfied with the answer after a long exchange: ‘I must understand this in order to remember’.*  *The supervisor gave feedback to a T6 that was supervising a T3 that the T6 should not have left her student unattended performing a procedure: ‘This is not to be interpreted as negative’.* |
|  | Unified inter-professional approach to teaching | *The doctor began the ward round by addressing and questioning the medical and nursing students together by name.*  *The biomedical scientist conducted a cardiac ultrasound on the ward, which lasted over one hour, during which she showed and explained every step to the medical students and student nurses.* |
|  | Supervisors’ motivation | *Supervisor: ‘Supervising is fun, then I am not just a nurse!’*  *Questioning the supervisors: when asked about reasons for working at the student ward, supervisors stated that their desire to teach and the ward’s reputation for a positive learning environment was the most important factor.* |
|  | Staff’s own learning | *Questioning the nurse supervisor: ‘When the doctors teach student nurses, the content is often aimed at us nurses also’.* |
| Student-dedicated space | Empowerment of learning in the student room | *Students asked ‘how’ or ‘why’ questions when in the student room, while the same scenarios in the nurse office had ‘yes/no’ or ‘which/what’ questions. Thinking-out-loud by students and staff was observed almost exclusively in the student room.* |
|  | A meeting point in a busy ward | *Questioning the students: T6 students felt secure knowing that their supervisor would be waiting in the student room for a debrief after an activity, and that they would not need to go searching the ward.* |
